# PD-L1 protein expression by Combined Positive Score (CPS) in patients with muscle invasive or advanced urothelial carcinoma: a single institution experience

**DOI:** 10.1186/s12885-023-11299-y

**Published:** 2023-09-01

**Authors:** Sarah Nasr, Fadi G. Haddad, Joseph Khazen, Joseph Kattan, Viviane Trak-Smayra

**Affiliations:** 1 Pathology Department, Hotel-Dieu de France University Hospital, Saint Joseph University, Beirut, Lebanon; 2 Hematology-Oncology Department, Hotel-Dieu de France University Hospital, Saint Joseph University, Beirut, Lebanon

**Keywords:** Urothelial carcinoma, PD-L1, CPS, 22C3 clone, Bladder cancer

## Abstract

**Introduction:**

Immune checkpoint inhibitors have revolutionized the treatment of patients with advanced urothelial carcinoma (UC) in the frontline and relapsed settings. Lebanon has one of the highest incidence of UC worldwide, yet no data exists regarding the expression of PD-L1 by Combined Positive Score (CPS) in advanced disease.

**Methods:**

We reviewed all patients treated at our institution for high grade UC, stage pT2 and above, between January 2017 and March 2021. We assessed the expression of PD-L1 by immunohistochemistry using 22C3 clone, and analyzed the association between PD-L1 expression and clinicopathological characteristics. PD-L1 positivity was defined as CPS score ≥ 10.

**Results:**

A total of 101 patients with advanced UC were included, with a median age of 71 years (range, 38 to 96 years); 78% were ever-smokers. Ninety-three of 101 patients (92%) had conventional UC and 43 patients (43%) had positive PD-L1 expression, with 12 patients having CPS of 100. The analysis by molecular subtype showed that patients with maximal CPS of 100 were enriched in “basal” molecular subtype. However, no association was found between PD-L1 expression (positive versus negative) and clinicopathological characteristics.

**Conclusion:**

The positivity of PD-L1 expression as assessed by CPS using the 22C3 clone in our population was almost comparable to the results reported in the occidental literature. Therefore, PD-L1 expression, as a potential predictor of response to immunotherapy, concerns the same percentage of the Lebanese UC patients.

## Introduction

The incidence of urothelial carcinoma (UC) in Lebanon is ranked the highest in the Middle East and in the world. In 2018, Lebanon reported the highest age-standardized rate (ASR) for bladder cancer (BC) in men and women, with 25.0 cases per 100,000 individuals [1]. During the last decade, frontline therapies for muscle-invasive or metastatic UC have relied primarily on cisplatin-based chemotherapies. More recently, in the era of immunotherapy, checkpoint inhibitors including anti-PD-1 and anti-PD-L1 agents, have been approved for the treatment of locally advanced or metastatic UC. Atezolizumab and pembrolizumab have been approved by the United States Food and Drug Administration’s (FDA) as a first-line therapy in patients ineligible for platinum-based chemotherapy and whose tumors have positive PD-L1 expression [2, 3]. However, the predictive role of PDL-1 for response to immunotherapy remains uncertain or inconclusive in other settings such as early stages or second-line and maintenance therapy in advanced stages [4].

In order to determine which patients would derive the most benefit from these therapies, the FDA and the European Medicines Agency (EMA) introduced “companion tests” that evaluate the immunohistochemical expression of PD-L1 within tumors cells (TC) and tumor infiltrating immune cells (IC) of the tumor microenvironment (TMI). Until now, the FDA has approved the use of four antibodies (22C3, 28–8, SP263 and SP142) with different methods for evaluating PD-L1 expression [5]. The prevalence of PD-L1 expression as detected by the 22C3 antibody using the CPS (Combined Positive Score) scoring method in locally advanced or metastatic UC in the Lebanese population is yet to be determined. The primary objective of our study is to assess this prevalence among patients diagnosed with muscle invasive or advanced UC in a Lebanese population.

## Material and methods

### Patient selection

Patients diagnosed with high grade UC, stage pT2 and above, and treated at Hotel-Dieu de France university hospital, with available Formalin-Fixed/Paraffin-Embedded (FFPE) specimens collected between January 2017 and March 2021 were included. Tumor specimens had to contain at least 100 viable tumor cells to ensure tissue adequacy.

Patients’ data was collected from computerized patient files and included age, sex, smoking status, clinical staging, estimated glomerular filtration rate (eGFR) and previous treatment when applicable (Bacillus Calmette-Guerin [BCG] treatment, chemotherapy and/or pelvic radiation therapy).

For all patients, a systematic histologic review was performed in order to confirm the initial diagnosis and determine histopathological criteria including the histologic variant as per the fourth edition of the World Health Organization (WHO) classification of tumors of the urinary system, histologic differentiation, molecular group when available (luminal, basal), histologic staging (pTNM) and group staging as defined by the 8^th^ edition of the American Joint Committee on Cancer (AJCC).

### Immunohistochemistry (IHC) with anti-PD-L1 antibodies (22C3 clone)

PD-L1 IHC was performed using the 22C3 pharmDX antibodies on Dako Autostainer Link 48, according to the provider technical manual, on 4-5 µm-thick tissue sections, using DAB detection system. For each case, two slides were performed: one with the PD-L1 antibody and one with a Negative Reagent Control (NRC). Each slide also contained a negative and a positive in-house control. Slides were independently evaluated by two trained pathologists. In case of discrepancy, a consensus was reached, or the mean value was adopted.

**Fig. 1:**
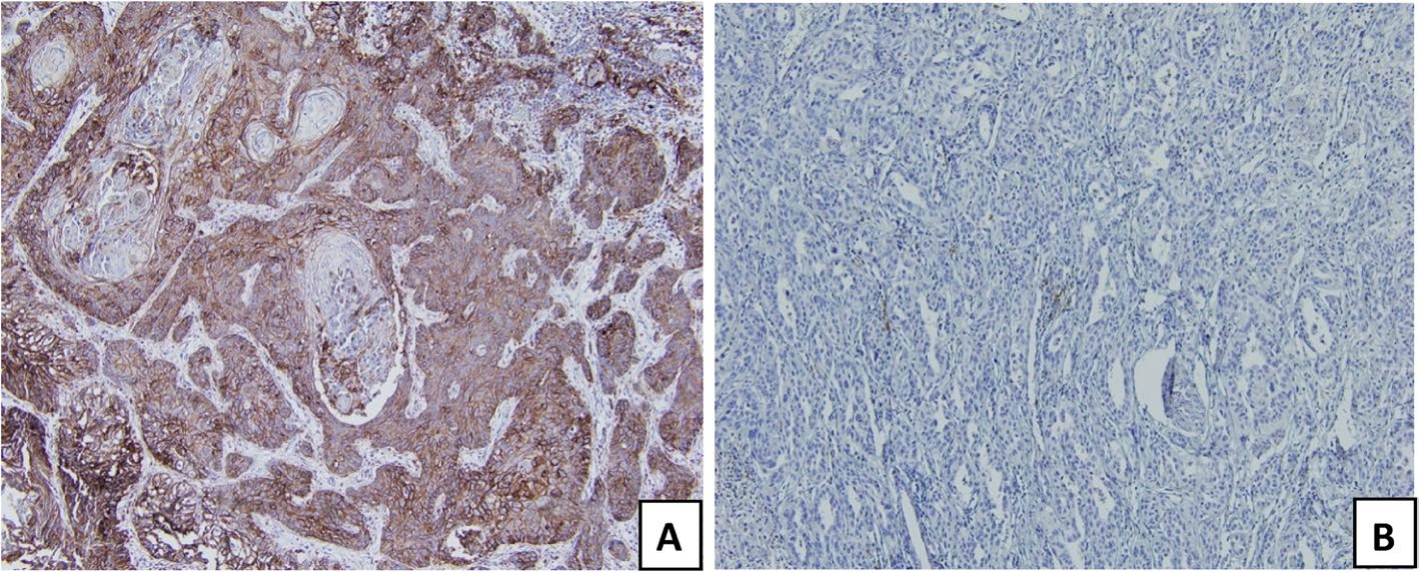
Immunohistochemical stainings showing **A** positive PD-L1 expression with CPS score of 100 and **B** negative PD-L1 expression with CPS score of 2 (× 100)

Controls were examined first, in order to invalidate any slide with unwanted staining. Slides were examined at × 200 magnification. We took into account any membranous staining in TC and any membranous and/or cytoplasmic staining and IC of TMI. Necrotic and apoptotic TC, plasma cells, neutrophils and fibroblasts were not evaluated. For UC, PD-L1 expression was interpreted using the CPS scoring system with a maximal value of 100:

A CPS equal to or greater than 10 was considered positive (Fig. 1) [6].

$$\mathrm{CPS}=\frac{\# PD-L1\,staining\,cells\,(tumors\,cells,\,lymphocytes,\,macrohages) }{Total\,\#\,of\,viable\,tumor\,cells}\times 100$$

### Statistical analysis

Continuous variables were described by medians and ranges while categorical variables were described by effectives (n) and percentages (%). PD-L1 score was analyzed both as a continuous variable (with a score value between 0 and 100) and as a categorical value (negative when inferior to 10 and positive when equal or superior to 10). Chi^2^ test was conducted to detect any correlation between two categorical variables. Wilcoxon Mann–Whitney test was used to compare continuous variables between two groups. The threshold for statistical significance was set for *P-value* < 0.05. Statistical analyses were performed using SPSS Statistics version 25.0 (IBM Corporation, New York, USA).

## Results

### Patient characteristics

A total of 101 patients with muscle invasive or advanced UCs were reviewed. The median age was 71 years (range, 38 to 96 years), 91 patients (90%) were age 60 years and older, and the majority were men (76%). Seventy-nine patients (78%) were either current or former smokers. Among the 101 patients, two had a previous history of occupational exposure to know carcinogens: one worked in the wood industry and the second in the metal industry. Both patients were also smokers. Twenty-five patients (25%) had an altered renal function with an estimated glomerular filtration rate < 50 mL/min. Thirty-nine patients (39%) had metastatic disease defined by involvement of lymph nodes beyond the common iliacs and/or non-lymph-node distant metastases. Twenty-one out of the 39 patients had lymph node metastases, 10 patients had lung metastases, seven patients had bone metastases, and 10 patients had involvement of other organs (Table 1).

**Table 1:** Patient characteristics

Characteristics	N (%) Median [range]
Age (years)	71 [38–96]
≥ 60 years	91 (90)
Male sex	77 (76)
Smoking status	
Current smoker	45 (47)
Former smoker	29 (31)
Non smoker	21 (22)
eGFR (mL/min)	77 [7–149]
< 50 mL/min	25 (25)
Stage group	
Stage II	48 (48)
Stage III	14 (14)
Stage IV	39 (39)
Prior BCG treatment	18 (18)
Prior chemotherapy	12 (12)
Prior radiation therapy	1 (1)

*Abbreviations*: *BCG* Bacillus Calmette-Guerin, *eGFR* Estimated glomerular filtration rate

### Tumor characteristics

Diagnosis of UC was established via trans-urethral resection in 52% of cases, surgical resection and cystectomies in 39% of cases, and sampling from metastatic sites in 6% of cases. Three cases were localized at the upper urinary tract and diagnosed following nephrectomy. Pathology examination revealed that 92% of tumors consisted of conventional UC and 8% were of special variants (six cases of poorly differentiated UC, one case of nested UC and one case of lymphoepithelioma-like UC). Forty-one cases presented with one or more differentiations, mainly squamous (25 cases), glandular (11 cases) and micropapillary (11 cases), sarcomatoïd (three cases), microcystic (one case), and independent cells differentiation (one case) (Table 2).

**Table 2:** Pathological characteristics of the tumors

Characteristics	N, (%)
**Pathology subtype**	
Conventional urothelial carcinoma	93 (92)
Special variant of urothelial carcinoma	8 (8)
**Inflexion/differentiation**	
Squamous	25 (25)
Glandular	11 (11)
Micropapillary	11 (11)
Sarcomatoid	3 (3)
Independent cells	1 (1)
Microcyctic	1 (1)
**Molecular subtype**	
Basal	26 (67)
Luminal	11 (28)

### PD-L1 expression

PD-L1 expression was evaluated by CPS score with reported values ranging from 1 to 100 with a median value of 7. Among 101 tumor specimens, 43 (43%) were found to have positive PD-L1 expression whereas 58 out of 101 (57%) had a CPS score inferior to 10 and were therefore classified as negative. Among the eight UC variants, five had a positive PD-L1 expression (63%). Twelve patients had an intense PD-L1 expression with a CPS score of 100. Of those twelve cases, seven had been analyzed for molecular subtype: six cases (86%) were of “basal” molecular subtype and one case (14%) was of “luminal” subtype.

### Correlation between PD-L1 expression and clinicopathological characteristics

We further analyzed the association between PD-L1 expression (positive versus negative) and clinical data (sex, smoking status, stage group, site of metastasis, eGFR, prior therapy) and histopathological and molecular characteristics (UC of specific variant, histologic differentiation, molecular group). No statistically significant association was found (Table 3).

**Table 3:** Association between PD-L1 expression and clinicopathological characteristics

	**PD-L1 expression**		***P***
	**Negative CPS (< 10%)** ***N* = 58** **n, (%)**	**Positive CPS (≥ 10%)** ***N* = 43** **n, (%)**	
**Age**			
< 71 years	22 (44.9)	18 (52.9)	0.376
≥ 71 years	27 (55.1)	16 (47.1)	
**Sex**			
Male	36 (73.5)	26 (76.5)	0.803
Female	13 (26.5)	8 (23.5)	
**Smoking status**			
Smoker	37 (78.7)	26 (83.9)	0.770
Non-smoker	10 (21.3)	5 (16.1)	
**Stage group**			
Stage II	22 (44.9)	10 (29.4)	0.307
Stage III	6 (12.2)	7 (20.6)	
Stage IV	21 (42.9)	17 (50)	
**Metastatic site**			
Lungs	6 (12.2)	3 (8.8)	0.731
Bones	5 (10.2)	2 (5.9)	0.695
Other	5 (10.2)	5 (14.7)	0.733
**Previous treatment**			
BCG (+)	11 (68.8)	7 (58.3)	0.698
BCG (-)	5 (31.3)	5 (41.7)	
Chemotherapy (+)	5 (33.3)	5 (41.7)	0.706
Chemotherapy (-)	10 (66.7)	7 (58.3)	
**Histologic type**			
Conventional UC	55 (95)	38 (88)	0.28
UC of special variant	3 (5)	5 (12)	
**Inflexion/histologic differentiation**			
Squamous	15 (26)	10 (23)	0.803
Glandular	8 (14)	3 (7)	0.187
Micropapillary	8 (14)	3 (7)	0.695
Sarcomatoid	0	3 (7)	0.165
With signet-ring cells	0	1 (2)	0.410
Microcystic	0	1 (2)	0.410
**Molecular subtype**			
Basal	14 (64)	12 (719)	0.467
Luminal	7 (32)	4 (23)	

*Abbreviations*: *BCG* Bacillus Calmette-Guerin, *UC* Urothelial carcinoma

## Discussion

Our study is the first to evaluate the prevalence of PD-L1 expression in muscle-invasive or metastatic UC, using the CPS scoring system, in Lebanon and the Middle-East. In a previous study, Mukherji et al. reported on 54 cases of muscle invasive urothelial carcinoma of conventional type only, evaluated for PD-L1 expression using the 5H1 clone, with a positivity threshold of 5% [7]. Only five tumors were found to be PD-L1-positive (9%). However, the differences in the scoring system, the positivity threshold and the antibody clone used in that study, preclude any direct comparison with our findings.

A positive PD-L1 expression with a CPS score ≥ 10 was found in 43% of our specimens. This was higher than the values described in the study by Faraj et al., using PD-L1 monoclonal antibody 5H1, reporting only 20% of muscle-invasive urothelial carcinoma expressing PD-L1 [8]. When the same CPS scoring method was used, similar levels of PD-L1 expression were observed across different studies. Bellmunt found a CPS score ≥ 10 in 31% of metastatic bladder cancer patients receiving pembrolizumab or chemotherapy as second-line therapy in the Keynote-045 [9]. Powels reported a CPS score ≥ 10 in 47% of patients with metastatic disease treated as first-line in the Keynote-361 [10].

In our population, a higher PDL-1 expression can be expected due to the high prevalence of smoking in 2020 [11], with 67% of smokers reporting water-pipe alone or in addition to cigarette [12]. However, literature review did not reveal a clear correlation between PD-L1 expression in bladder cancer and tobacco consumption, but revealed some correlation between smoking and the effectiveness of immune checkpoint inhibitors when ever-smokers were compared to never-smokers [13].

The discordance in PD-L1 expression in the literature could be explained by the absence of standardized method for evaluation. Reis et al. reported a 30% positivity rate of PD-L1 in conventional UC using the 22C3 clone when accounting only for positive TC. However, when IC were taken into account, the prevalence of PD-L1 rose to 39% in that same population using the same antibody clone [14]. These results are concordant with our findings, considering that 40% of our conventional urothelial carcinoma are PD-L1 expressors.

Previous studies revealed that urothelial carcinomas with special differentiations were more likely to express PD-L1 [14, 15] (p). In our cohort, 63% (five out of eight cases) of our variants had a positive CPS score. However, given the small sample size, no statistically significant correlation was found. In a trial of 195 patients with advanced UC treated with atezolizumab in the second-line setting, a higher prevalence of PD-L1 expression was observed in patients with “basal” molecular subtype compared with their “luminal” counterpart (60% versus 23%, *P* < 0.001) [16]. In our cohort, despite the small sample size, “basal” molecular subtype was enriched in patients with maximal CPS score. These findings suggest that tumors with “basal” molecular subtype might be more responsive to treatment with immune checkpoint inhibitors.

## Conclusion

The positivity of PD-L1 expression as assessed by CPS using the 22C3 clone in our population of patients with advanced UC was almost comparable to the results reported in the occidental literature. Therefore, PD-L1 expression, as a potential predictor of response to immunotherapy, concerns the same percentage of the Lebanese UC patients. Thus, our population deserves “the luxury” to access immunotherapy for urothelial tumors and avoid any cancer treatment disparities [17].

## Data Availability

Data may be shared to qualified researchers upon reasonable request to the corresponding author. No identifying data will be provided.
